# Case Report: Metagenomics Next-Generation Sequencing for Diagnosing Cerebral Infarction and Infection Caused by Hematogenous Disseminated Mucormycosis in a Patient With Acute Lymphoblastic Leukemia

**DOI:** 10.3389/fmed.2021.779981

**Published:** 2021-12-17

**Authors:** Bingbing Wen, Lisheng Cai, Yun Cai, Xin Du

**Affiliations:** Department of Hematology, The Second People's Hospital of Shenzhen, The First Affiliated Hospital of Shenzhen University, Shenzhen, China

**Keywords:** mucormycosis, metagenomics next-generation sequencing, *Rhizomucor miehei*, cerebral infarction, cerebral infection, hematogenous disseminated, leukemia

## Abstract

Disseminated mucormycosis, a serious complication, is associated with high mortality in patients with acute leukemia after chemotherapy. Blood cultures are always negative because of recurrent empirical antifungal treatments. The identification of pathogens is important for diagnosis and therapy. In this case report, we diagnosed culture-negative disseminated mucormycosis with *Rhizomucor miehei* infection leading to cerebral infarction in a patient with leukemia using metagenomics next-generation sequencing (mNGS) form peripheral blood, cerebral spinal fluid, and bronchoalveolar lavage fluid. mNGS technology can be applied to precisely diagnose culture-negative disseminated mucormycosis.

## Introduction

Disseminated mucormycosis, characterized by necrosis and infarction of tissues due to invasion of vasculature by hyphae, is a rare disease that commonly occurs in severely immunosuppressed patients (1, 2). Disseminated mucormycosis is associated with a high mortality rate of approximately 96% in patients with acute leukemia after chemotherapy (3–5). Positive culture and histological evidence for mucormycosis from blood or tissue biopsies are the key diagnostic criteria (6–8). However, positive blood culture results perform poorly at the onset of clinical symptoms and change of videography suspected to be mucormycosis (9–13). The positive culture rate is only 38% for cerebral mucormycosis (14) and 50% for all types of mucormycosis (3). Besides, biopsy specimen is not always feasible in most vulnerable populations. Therefore, molecular diagnostic technology as an adjuvant method are used to improve the early identification of the causative pathogens and guide treatment for patients.

Metagenomic next-generation sequencing (mNGS), a new molecular diagnostic technology, detects microbial nucleic acids with genomics method contained in samples. Its most significant advantage is the lack of requirement for culture and premise hypothesis (14, 15). mNGS has been applied in infectious diseases including mucormycosis to identify causative pathogens (16, 17). We present a case of culture-negative mucormycosis with *Rhizomucor miehei* infection leading to cerebral infarction diagnosed by mNGS of peripheral blood (PB), cerebral spinal fluid (CSF) and bronchoalveolar lavage fluid (BAL).

## Case Description

**Figure 1:**
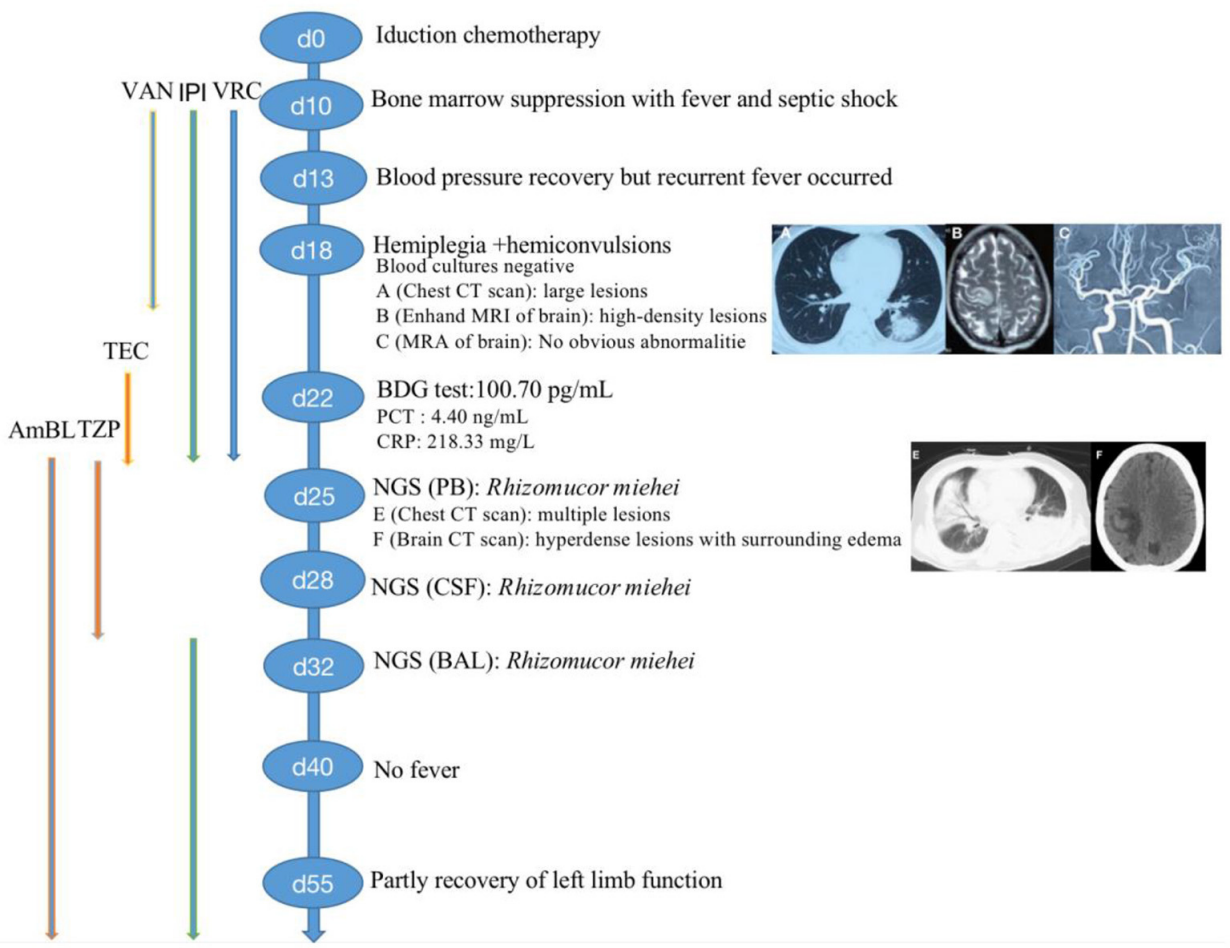
Clinical course of the patient (schematic). BDG, (1,3)-b-D-glucan; PCT, procalcitonin; CRP, c-reactive protein; CT, computed tomography; MRI, magnetic resonance imaging; MRA, magnetic resonance angiography; VRC, voriconazole; IPI, Imipenem; VAN, Vancomycin; TEC, teicoplanin; AmBL, liposome-associated amphotericin B; mNGS, metagenomics next-generation sequencing; PB, peripheral blood; CSF, cerebral spinal fluid; BAL, bronchoalveolar lavage fluid.

A 44-year-old man with acute lymphoblastic leukemia (ALL) underwent induction chemotherapy (IC) (day 0). An outline of the episodes is showed in Figure 1. Bone marrow suppression with fever and septic shock occurred on day 10. Patient suffered serious infection, and empirical treatments were used with imipenem (IPI, 1 g, every 8 h, intravenous injection), vancomycin (VAN, 1 g, every 12 h, intravenous injection) and voriconazole (VRC, 200 mg, every 12 h, intravenous injection) as broad-spectrum antibacterial and antifungal prophylaxis and empirical treatments were used with broad-spectrum antibiotics including antifungal prophylaxis. The patient's blood pressure recovered but recurrent fever occurred after 3 days. At that time, a series of cultures of peripheral blood (PB) were negative from day 10 to day 28. No pathogen could be detected, and the patient experienced neutropenia from day 10 to day 25 (Figure 1). However, hemiplegia and hemiconvulsions suddenly occurred on patient's right limb, and computed tomography (CT) scans of patient's brain showed a hyperdense lesion with surrounding edema, which was highly suspected as cerebral infarction in the right parietal lobe and small hypodense areas in the left and right parietal lobes. No obvious abnormality was showed by magnetic resonance angiography (MRA) of patient's brain, but CT scans of the lung showed multiple hyperdense lesions on day 18 (Figure 1). Febrile neutropenia in patients after chemotherapy with cerebral symptoms may be highly indicative of infections in the brain (18, 19).

**Figure 2:**
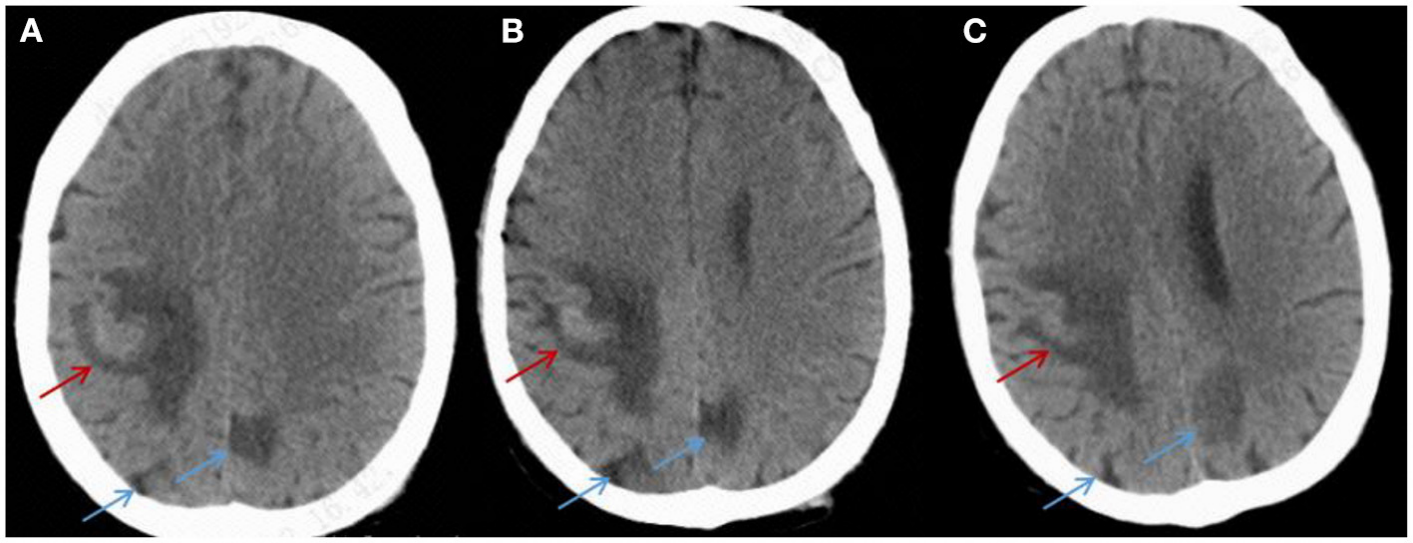
CT imaging for the patient's brain. **(A)** On day 25, a CT scan of the brain showed hyperdense lesions with surrounding edema in the right parietal lobe (red arrow) and hypodense areas (blue arrow) in the left and right parietal lobes. **(B,C)** After 2 and 3 weeks of treatment, a CT scan showed that the lesions were absorbed in the left and right brain (red arrow and blue arrow).

**Figure 3:**
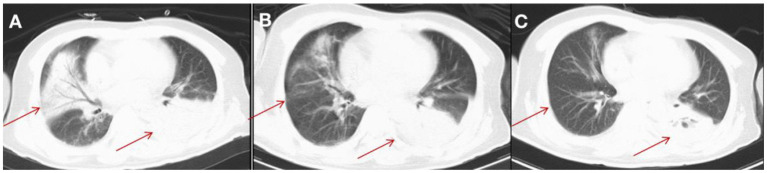
CT imaging for the patient's lung. **(A)** On day 25, a CT scan of the lung showed multiple inflammatory lesions (red arrow) and small amount of bilateral pleural effusion in both lungs. **(B)** After 2 weeks of treatment, a CT scan showed that the lesions were absorbed in the right side of patient's lung. **(C)** After 3 weeks of treatment, a CT scan showed that lesions were significantly absorbed in the right side of patient's lung (red arrow) and smaller amount of bilateral pleural effusion in the side of patient's lung than that after 2 weeks of treatment.

**Figure 4:**
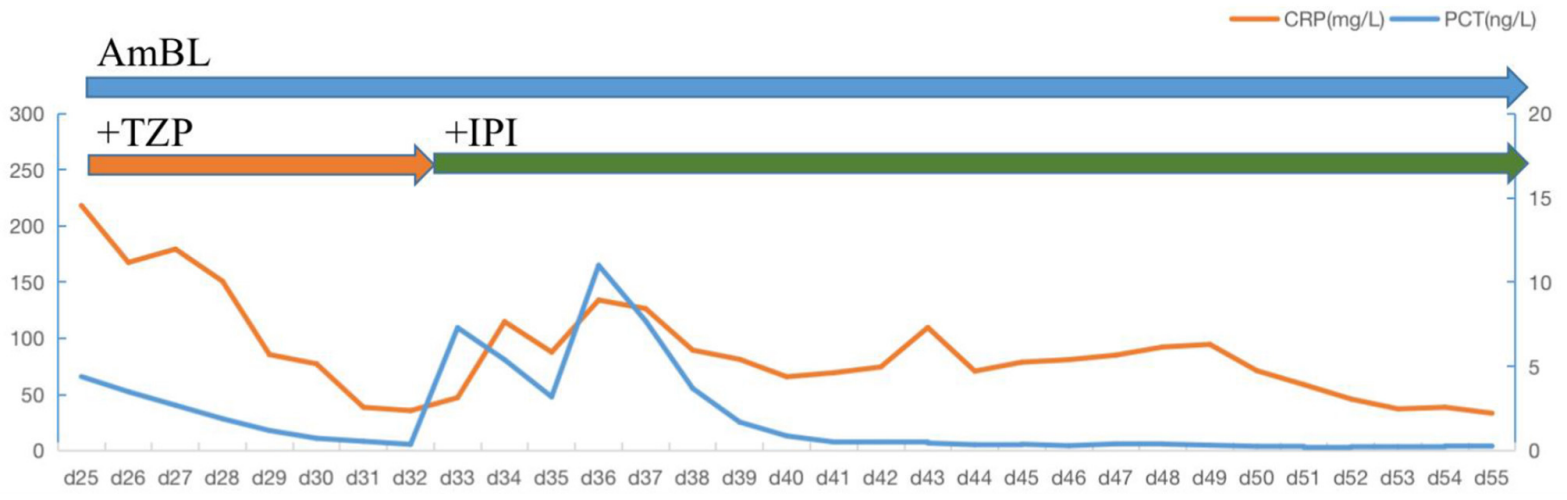
Daily course of the patient's treatment. Horizontal thick blue lines show the medications administered; VRC, voriconazole; AmBL, Liposome-associated amphotericin B; TZP, piperacillin-tazobactanm; IPI, Imipenem; PCT, procalcitonin; CRP, C-reactive protein.

Treatment was continued VRC and IPI, and changed to teicoplanin (TEC, 400 mg, daily, intravenous injection) as antibacterial and antifungal drugs Treatment was changed to voriconazole (VRC) and imipenem as antifungal and antibacterial methods after a positive result for the test of (1,3)- β-D-glucan (100.70 pg/mL, Guangzhou Zhaokang Biotechnology Co., Ltd) on day 22. However, the fever was unresponsive and the lesions in the brain and lung were more serious a week later. The procalcitonin (PCT) level was elevated to 4.40 ng/mL, and c-reactive protein (CRP) reached 218.33 mg/L. Aminoleucine transferase (ALT) and aspartate transaminase (AST) reached 145 U/L and 242 U/L, respectively. Other drugs were applied to protect patient's liver function. *Rhizomucor miehei* infection was shown by mNGS (Genskey Medical Technology Co., Ltd, Beijing, China. NextSeq 500) of PB with high relative abundance about 99.94% on day 25 (Table 1), and liposome-associated amphotericin B (AmBL, 100 mg, daily, intravenous injection) was immediately used as antifungal therapy from day 25 to day 55. The patient's body temperature returned to normal (36–37°C) after 3 days of treatment and his complete blood count (CBC) recovered from neutropenia on day 28. Infection with *Rhizomucor miehei* was also proven by mNGS both in CSF with relative abundance about 0.35% on day 28 and BAL with relative abundance about 28% on day 35 (Table 1), and the routine and biochemical examinations of CSF were negative (Table 2). After 2 and 3 weeks of antifungal treatment, CT scans showed that lesions were obviously absorbed both in the brain (Figure 2) and the lung (Figure 3), and PCT and CRP were also recovered following antifungal therapy (Figure 4). Finally, the patient's general condition improved, and his right limb function partly recovered on day 55. He requested to go back to the local hospital to continue antifungal therapy due to his family reasons.

**Table 1:** Pathogenic microorganisms detected in peripheral blood, cerebral spinal fluid, and bronchoalveolar lavage fluid specimen of patient by next-generation sequencing.

**Genus**			**Species**	
**Name**	**Sequence number**	**Relative abundance (%)**	**Name**	**Sequence number**
**1.Peripheral blood**				
Rhizomucor	41500	99.94	*Rhizomucor miehei*	40895
**2. Cerebral spinal fluid**				
Rhizomucor	1	0.35	*Rhizomucor miehei*	1
**3. Bronchoalveolar lavage fluid**				
Rhizomucor	7	28.00	*Rhizomucor miehei*	7
Aspergillus	13	52.00	*Aspergillus flavus*	4
Aspergillus	13	52.00	*Aspergillus terreus*	4
Elzabethkingia	125838	94.66	*Elzabethkingia anophelis*	85818
Stenotrophomonas	2511	1.89	*Stenotrophomonas maltophilia*	2404

**Table 2:** Cerebrospinal fluid test on day 28.

**Parameter (unit)**	**Result**	**Normal range**
Colors	Transparent	Transparent
Pandy test	Negative	Negative
Red blood cell (10^∧^6/L)	0	N/A
Wite blood cell (10^∧^6/L)	0	(0-8) x 10^∧^6/L
Glucose (mmol/L)	3.10	2.5–4.5
Chloride (mmol/L)	126.3	120–132
CSF protein (g/L)	374.5	150–450
Tumor cell	Negative	Negative

After 2 months of follow-up by telephone, we learned that patient changed to receive antifungal treatment with amphotericin B (AmB, 150 mg, daily, intravenous injection) and posaconazole (Pos, 300 mg, daily, orally) for other 2 months, because patient's liver had recovered to normal function. Besides, significant reduction in the size of the lesions on imaging tests of this patient were reported. The next chemotherapy for ALL was also prepared to carried out. The antifungal drug regimen was well-tolerated and achieved a remarkable effect.

## Discussion

Mucormycosis occurred during the period of neutropenia after chemotherapy for ALL in this patient. Cerebral infarction and fungal encephalitis are caused by hematologic dissemination with poor outcomes. Only a few case reports on disseminated mucormycosis have been reported, and cerebral manifestations of mucormycosis have been recorded even less frequently (20–22).

The high mortality rate in patients with disseminated mucormycosis necessitates rapid diagnosis (3–5). However, very low positive rates of blood cultures from patients with ALL after chemotherapy were observed due to empirical antifungal therapy (23). Pagano, L. et al. showed that only 4 leukemia patients (~10%) were diagnosed ante-mortem among 116 patients with recorded infections of pulmonary filamentous fungal (24). Another study showed that approximately 35% of 37 patients with histologically recorded mucormycosis and hematological malignancies ware diagnosed ante-mortem (20). CT scans of the lung and the brain provide significant information's for the diagnosis of invasive fungi (25, 26). However, it is difficult to distinguish the kind of fungal infections by these imaging methods.

mNGS, a high-throughput screening method, is an emerging and relatively effective diagnostic method to detect pathogens that are culture-independent, especially for non-cultivable or uncommon microbiological organisms (27, 28). In addition, mNGS is used to detect pathogens by sequencing of extracted DNA from specimens, which is a non-culture-based and fast method (29). In this case study, mNGS of PB, CSF, and BAL was well employed to diagnose disseminated mucormycosis infection leading to cerebral infarction. In addition, high sequence of elizabethkingia and stenotrophomonas was detected by mNGS of BAL later. It could be a small amount of bacteria hidden in the infusion port, which caused the patient's second infection. Infusion port of patient was removed, and IPI was applied immediately again. PCT and CRP was controlled later.

In general, early initiation of antifungal therapy improves the outcome of mucormycosis infection. A previous study showed that 70 patients with hematologic malignancy and infection of mucormycosis who experienced delayed amphotericin B therapy (starting therapy more than 6 days after diagnosis) were reported to have two-fold increase in mortality rate at 12 weeks after diagnosis compared to early treatment (82.9% versus 48.6%) (30). In the present case study, mNGS, a fast and non-culture-dependent method, was used to diagnose mucormycosis infection, and the initial therapy of AmBL was performed immediately to treat this disease. The patient's general condition improved, and he eventually partly recovered his left limb function.

mNGS technology, with the advantages of speed and precision, is a selective method for culture-negative diagnosis of the fungal infection, and it can be applied to identify microbes in other symptoms in the future. Besides, some factors such as warm ischemia can interpreted NGS data, which may be the obstacles of NGS. In addition, febrile neutropenia in patients after chemotherapy with cerebral symptoms may be highly indicative of infections in the brain.

## Conclusion

mNGS testing of PB, CSF, and BAL is a reliable and fast method for precisely diagnosing culture-negative disseminated mucormycosis. Early identification of infectious pathogens can guide antifungal therapy in such patients.

## Data Availability Statement

The original contributions presented in the study are included in the article/supplementary materials, further inquiries can be directed to the corresponding author/s.

## Ethics Statement

The studies involving human participants were reviewed and approved by Second People's Hospital of Shenzhen. The patients/participants provided their written informed consent to participate in this study. Written informed consent was obtained from the individual(s) for the publication of any potentially identifiable images or data included in this article.

## Author Contributions

XD contributed to conception and design of the study. LC and YC analyzed and collected data. BW drafted the manuscript. All authors contributed to interpretation of the results, revised the manuscript, and read and approved the final manuscript.

## Funding

This study was supported by Shenzhen Key Medical Discipline Construction Fund (SZXK008).

## Conflict of Interest

The authors declare that the research was conducted in the absence of any commercial or financial relationships that could be construed as a potential conflict of interest.

## Publisher's Note

All claims expressed in this article are solely those of the authors and do not necessarily represent those of their affiliated organizations, or those of the publisher, the editors and the reviewers. Any product that may be evaluated in this article, or claim that may be made by its manufacturer, is not guaranteed or endorsed by the publisher.

## Abbreviations

ALL: Acute lymphoblastic leukemia
AmBL: Liposome-associated amphotericin B
BAL: Bronchoalveolar lavage fluid
CSF: Cerebral spinal fluid
CT: Computed tomography
mNGS: Metagenomics next-generation sequencing
PB: Peripheral blood
VRC: Voriconazole.
